# The long-term Effects of the Coronavirus Disease (COVID-19) on Auditory Thresholds: A Case-Control Study

**DOI:** 10.22038/ijorl.2024.80236.3698

**Published:** 2025

**Authors:** Sadegh Jafarzadeh, Saeid Eslami

**Affiliations:** 1 *Department of Audiology, School of Paramedical and rehabilitation Sciences, Mashhad University of Medical Sciences, Mashhad, Iran.*; 2 *Department of Medical Informatics, Faculty of Medicine, Mashhad University of Medical Sciences, Mashhad, Iran.*

**Keywords:** Hearing loss, COVID, Long-COVID symptom, Sensorineural hearing loss

## Abstract

**Introduction:**

The available evidence about hearing loss in Coronavirus disease (COVID-19) patients mostly show sensorineural hearing loss, and the long-term effects of COVID-19 on auditory thresholds are unknown. This study aimed to compare the auditory threshold results in COVID-19 patients (several months after infection) with a control group.

**Materials and Methods:**

The clinical diagnosis of COVID-19 was confirmed with positive polymerase chain reaction findings and radiology images. Hearing evaluation was performed with an audiometry test and a calibrated audiometer in a sound-treated room. The results of 177 patients were compared with those of the 589 matched control group. In both groups, subjects over 50 years old or with any history of ear disease were excluded from the study.

**Results:**

The time interval between infection with COVID-19 and hearing tests was 170.51±98.38 days. There was no significant difference between the auditory thresholds in different frequencies in both groups. Also, no significant difference was observed between the auditory thresholds of the two groups in the first, second, and third trimesters after being infected.

**Conclusion:**

This study did not show the long-term effects of COVID-19 on auditory thresholds, and the findings do not support hearing loss as a long-term COVID symptom.

## Introduction

The pandemic of Coronavirus disease (COVID) had many effects on various aspects of people's health. A large number of people got infected with COVID worldwide. There were multiple reports of hearing loss among COVID-19 patients. The first report of hearing loss in COVID patients was presented on March 15, 2020, and in this report, persistent sensorineural hearing loss (SNHL) in an older woman was reported (1), which was persistent even after recovery from the disease. 

The subsequent reports indicated hearing loss in some patients with COVID-19, most of which were related to sudden SNHL (2-7). In one case, a patient was diagnosed with otitis media (8). In research on 20 patients with COVID and comparing them with the control group, more SNHL in the high-frequency range was observed in the patient group (9). A questionnaire examination of hearing loss handicapping also showed a significant difference between COVID-19 patients and those in the control group (10). 

However, earlier studies had some problems, and some patients were susceptible to presbycusis. Also, the timing of the development of hearing loss with COVID and the presence of accompanying symptoms such as dizziness, tinnitus, and earache were less noticed. These studies evaluated the post-COVID symptoms of hearing loss, and few researchers have investigated the long-term and chronic effects of hearing loss in COVID patients. Although some cases of hearing loss have been observed in COVID patients, these reports, mostly related to post-COVID and chronic or long-term COVID-19, have been less investigated. Investigating chronic changes is important for two reasons: Hearing is a main sense that is very important to our daily lives. Even in adults, chronic hearing loss can cause numerous communication, psychosocial, and work-related difficulties. 

Hearing loss is related to the reduction of health-related quality of life, and its treatment is related to its improvement (11). The second issue is the possible lack of knowledge about hearing loss in some patients. This issue is also observed in other diseases, such as presbycusis and noise-induced hearing loss. This issue is more frequent in gradual or high frequencies SNHL. The ability to notice hearing loss in the patient depends on many internal and external factors. This study aimed to compare the results of auditory thresholds in COVID-19 patients (several months after being infected) with those of a control group.

## Materials and Methods

It was a cross-sectional study in which the auditory thresholds of COVID-19 patients were compared with the matched control group. In the patients’ group, the clinical diagnosis of COVID-19 was confirmed by polymerase chain reaction (PCR) test and radiological imaging. The control group included Mashhad University of Medical Sciences employees with the same age conditions as the patients. The selection of patients and the control group was based on Poisson random sampling for six months from December 2021 to May 2022. The study was approved by the ethics committee of Mashhad University of Medical Sciences (IR.MUMS.REC.1400.210).

Both patient and control groups went to the Persian Cohort Center for hearing evaluations, which were examined using pure tone audiometry (PTA) and tympanometry tests. ASHA method was used for PTA to determine the thresholds of 250 to 8000Hz. Before performing the test, subjects were trained and instructed on how to respond. The tests were performed in a sound-treated room with a calibrated audiometer. 

The examiner, instruments, method, location, and evaluation conditions were the same for all subjects. The results of tympanometry were used to evaluate the condition of the middle ear and contribute to the diagnosis of hearing loss. 

In the selection of patients, three principles were considered: 1- confirmation of the disease diagnosis with PCR tests and radiological imaging, 2- temporal concordance between the onset of COVID-19 and the development of hearing loss by removing all cases with a previous history of hearing loss, and 3- the absence of other previous ear diseases such as presbycusis (subjects over 50 years of age), noise-induced hearing loss, otosclerosis, otitis media, ototoxicity, and Meniere's disease. All subjects who did have these conditions were excluded from the study. 

These subjects were excluded from the study so we could evaluate the long-term effects of COVID-19 on hearing (12).

### Data analysis

Mean and standard deviation were used to describe the quantitative data. The Kolmogorov-Smirnov test was used to check the normal distribution. Due to the normal distribution, the results of different frequencies were compared with the t-test. The results of three trimesters after infection and the control group were compared with the ANOVA test. The Tukey post-hoc test was used to evaluate the significance of differences between the three trimesters and the control group. 

## Results

In this study, the auditory thresholds of 177 patients were compared with 589 controls. The ages of the patients and control group were 41.18±4.99 and 41.87±3.59 years, respectively. The two groups had no significant age difference (p> 0.05). 119 (67.2%) patients and 223 (37.9%) control group members were female. The auditory thresholds in all frequencies were compared in two groups (Table 1).

**Table 1:** the mean and standard deviation of auditory thresholds in COVID patients and control group.

**Group**	**Ear**	**250Hz**	**500Hz**	**1000Hz**	**2000Hz**	**4000Hz**	**8000Hz**
Patients	R	18.50±8.403	22.43±7.368	17.40±7.633	15.00±10.155	17.66±12.580	18.53±15.359
	L	18.84±7.955	22.18±7.284	16.07±7.667	13.16±9.528	16.95±13.251	18.76±15.654
Patients-First trimester	R	15.68±6.142	20.54±4.531	16.62±5.144	12.16±7.124	16.49±13.169	15.95±14.522
	L	15.14±5.202*	19.19±3.635	15.00±5.000	11.35±6.197	14.32±11.497	14.73±11.604
Patients-Second trimester	R	18.62±10.771	22.92±9.957	18.31±10.506	16.46±13.367	18.62±15.143	19.62±16.963
	L	19.92±10.698	22.92±9.917	16.54±10.859	14.08±12.371	19.46±15.839	20.31±17.851
Patients-Third trimester	R	19.80±6.599	22.93±5.581	17.00±5.390	15.13±7.754	17.40±9.599	18.87±14.322
	L	19.73±5.446	23.00±5.390	16.20±4.989	13.27±7.905	16.07±11.249	19.40±15.200
Control	R	19.03±7.671	22.72±7.343	18.26±7.207	16.13±9.051	20.59±12.699	20.17±16.231
	L	19.75±6.927	21.73±6.459	16.53±6.735	14.24±9.183	18.80±12.384	19.13±15.478
*Significant difference with the control group at ˃ 0.05 **Significant difference with the control group at ˃ 0.01

The results of patients and control groups were compared with a t-test. The two groups had no significant difference in any frequency (250 to 8000Hz). 

The time interval between hearing tests and COVID-19 infection was 170.51 ± 98.38 days. 37 (20.9%), 65 (36.7%) and 75 (42.3%) patients were evaluated in the first to third trimester after infection, respectively. 

For evaluating the possibility of temporary hearing loss in patients, the results of each trimester were compared with the control group separately. These results also showed no significant difference between the control group and the three trimesters (Table 1). 

The Tukey Post-hoc showed that only the 250Hz thresholds for the left ear in the first trimesters were significantly better in patients than in the control group. However, the difference was very small and is not clinically important.

## Discussion

The current study showed the lack of long-term effects of COVID-19 on auditory thresholds. There was no sudden SNHL or temporary hearing loss in the examined sample. These findings are important because a large number of people in our country, as well as the whole world, were infected with COVID, and the absence of residual hearing loss is promising. Hearing loss after COVID-19 has been documented in numerous case reports, original studies, and reviews (3,4,13,14), with many of these studies identifying post-COVID sudden SNHL. 

The affected ear varied across studies, with different degrees of hearing loss reported. Some of the early studies indicated that high-frequency SNHL can occur as a post-COVID symptom. However, these studies faced objections due to improper COVID diagnosis, temporal non-concordance between infection and hearing loss onset, and failure to exclude prior otologic disorders (12). Also, sudden COVID-19-related SNHL has a low incidence rate (0.2-7.6%) (14). some theories have been proposed regarding permanent hearing loss in COVID patients, such as the possibility of the effects of COVID-19 on the auditory cortex (15) or a hypoxic state in the auditory centers (16). In these post-COVID studies, hearing loss is usually diagnosed shortly after the onset of the disease. 

The COVID diagnosis was confirmed through PCR tests and imaging in the present study, and temporal concordance was maintained while excluding prior otologic disorders. Also, the time interval between the diagnosis and the time of hearing evaluation is long, and no permanent hearing loss was observed in our patients compared to the control group. This finding aligns with a review article that confirms hearing loss as a post-COVID symptom but does not categorize it as a long-COVID symptom (17). 

Other studies focused on long-COVID did not recognize hearing loss as a related symptom (18,19). Conversely, one review article does identify hearing loss as a symptom of long-COVID (20). This review refers to two studies: one that utilized an online survey without a PTA test (21) and another that assessed post-COVID symptoms rather than long-COVID symptoms (22).

One weakness of the present study is that it uses a cross-sectional design. A longitudinal and cohort study seems more suitable for evaluating long-term COVID-19 symptoms. However, due to our limitations, the cross-sectional design was selected. 

## Conclusion

The current study showed that COVID-19 had no long-term effects on our patients’ auditory thresholds.
